# Sub-ppm-Level Ammonia Detection Using Photoacoustic Spectroscopy with an Optical Microphone Based on a Phase Interferometer

**DOI:** 10.3390/s19132890

**Published:** 2019-06-29

**Authors:** Oscar E. Bonilla-Manrique, Julio E. Posada-Roman, Jose A. Garcia-Souto, Marta Ruiz-Llata

**Affiliations:** Electronics Technology Department, Carlos III University of Madrid, 28911 Leganés, Spain

**Keywords:** photoacoustic spectroscopy, gas detection, optical microphone, resonant cell, membrane

## Abstract

A sensitive optical microphone for photoacoustic spectroscopy based on the common path topology of a fibre laser Doppler vibrometer (FLDV) using phase-generated carrier demodulation and a slim diaphragm as an acoustic wave transducer was demonstrated. A resonant gas cell was adapted to enhance gas-detection performance and simultaneously provide efficient cancellation of the window background acoustic signal. Ammonia (NH_3_) was selected as the target gas. The absorption line was experimentally identified using a distributed feedback laser diode emitting at 1530 nm. The linearity and sensitivity of the gas sensor were measured using wavelength modulation spectroscopy with second harmonic detection. A Teflon diaphragm was used to implement the optical microphone, along with the FLDV, showing a minimum detectable pressure of 79.5 μPa/Hz^1/2^. The noise-equivalent absorption sensitivity for NH_3_ detection at the absorption line at 1531.7 nm was 1.85 × 10^−8^ W cm^−1^ Hz^−1/2^, and the limit of detection was 785 ppbv.

## 1. Introduction

Photoacoustic spectroscopy (PAS) has been used in different applications for trace gas-sensing due to several features, such as high sensitivity, high selectivity, low cost, linear response and measuring system compactness. It has been employed as an analysis technique with potential application in microbiology, physiology, health diagnostics and environmental gas monitoring [1,2]. PAS is based on the detection of acoustic pressure waves (sound) that are generated due to molecular absorption of modulated optical radiation [3]. A classical experimental setup for PAS involves a monochromatic light source, whose amplitude or wavelength is modulated, and a non-resonant or resonant gas cell with a pressure-sensitive detector. Typically, the pressure detector is a conventional sensor (condenser microphone or piezoelectric microphone), but other detection schemes, such quartz-enhanced photoacoustic (QEPAS), have gained a lot of attention due to the increase of sensitivity combined with small sampling volumes [4]. However, these techniques based on electronic transducers have limitations for industrial monitoring, where trace gas sensors for hazardous substances in hostile environments are of increasing importance. Electronic transducers might be affected by metal corrosive substances and by electrical interferences. As an alternative, sound detection via optical methods in combination with a gas cell manufactured with high chemical resistance materials can be used for gas detection devices in hazardous environments or where humidity, high temperature or strong electromagnetic interferences are presented. This technology widens the range of applications for PAS systems and can also reduce their maintenance costs and improve their performance.

High sensitivity optical microphones and pressure sensors have been developed based on a diaphragm placed in close proximity to a cleaved end of an optical fibre, thus forming a Fabry–Pérot interferometer (FPI). As the sensitivity of these optical microphones depends on the size and thickness of the diaphragm membrane, different materials, such as silver [5], silicon [6] or graphene [7] have been used for sensor miniaturization. PAS systems using these kinds of optical microphones have been reported in the literature [8,9]. Miniature PAS systems have also been proposed by taking advantage of using the optical microphone hollow cavity as the gas cell and using the optical microphone fibre to guide the absorption excitation wavelength [10]. Another approach for all-optical PAS systems is using a silicon cantilever for acoustic detection, as the displacement of a cantilever is more linear and about two orders of magnitude larger than that of a conventional membrane with similar dimensions [11]. In cantilever-enhanced photoacoustic spectroscopy (CEPAS), introduced by Wilcken and Kauppinen [12], the acoustic signal is detected by measuring the movement of the cantilever using a Michelson-type laser interferometer. Many studies have reported good performance with CEPAS systems [12,13,14]. Miniaturized PAS systems based on this approach have also been developed by placing the cantilever transducer on top of a glass ferrule [15] and by using micro-machined devices [16], obtaining performance parameters comparable to those reported for bulkier PAS analysers. These last two systems incorporate one fibre for the absorption excitation and another fibre to build an FPI with the cantilever for interferometric readout of the cantilever displacement.

FPI and Michelson-type interferometric readings of acoustic transducers typically measure the amplitude of the interference signal using optical detectors. For that purpose, the interferometer should be set to the point of highest sensitivity corresponding to a linear response region with the largest slope in the interference fringe, which suggest tuning the laser wavelength and continuous calibration as the output depends on the fringe visibility [17]. In this paper, we report the implementation of a PAS system based on a standard tube resonant gas cell where two types of acoustic detectors have been placed. The first consists of a thin polymer diaphragm placed in the centre of the tube that acts as the acoustic transducer whose displacement is measured using a fibre laser Doppler vibrometer (FLDV) [18]. The second is an electrical microphone used for comparison purposes. The proposed FLDV is a phase interferometer that does not require laser stabilization or path length tuning, simplifying the transducer readout system. A common path topology (CPT) removes the disturbance onto the fibre lead. The PAS system was tested for NH_3_ by means of a diaphragm with a minimum detectable pressure of 79.5 *μ*Pa/Hz^1/2^, obtaining a minimum detection limit of 785 ppb and an NNEA = 1.85 × 10^−8^ W cm^−1^ Hz^−1/2^.

## 2. Materials and Methods

### 2.1. Gas Cell and Acoustic Transducer Design

**Figure 1:**
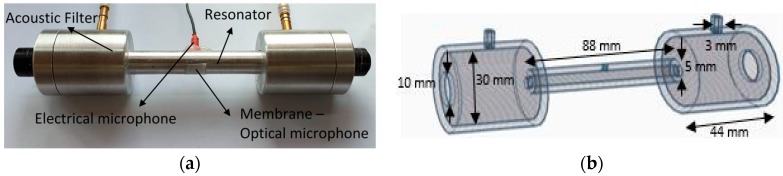
Resonant gas cell (RGC). (**a**) Metal resonant gas cell. (**b**) Structure and dimensions of the resonant gas cell (in mm).

The target gas is confined inside a gas cell, where the photoacoustic wave is generated. The reference cell design used in this study is shown in Figure 1. It is a resonant cell that consisted of a cylinder resonator (a pipe), with a 5-mm diameter (*r* = 2.5 mm) to allow the laser beam to pass through the resonator. It was terminated with two cylindrical buffer volumes, which are considered acoustic filters where the gas inlet/outlet connectors and the optical windows were installed. It is based on a previous design [19] with two main modifications; the first is the building material, it was made of aluminium, and the other was including a second hole (3-mm diameter) in the centre of the resonator for installing a diaphragm.

Equation (1) shows the resonance frequency of a cell open on both sides [20] and with two holes:

(1) $$fr=\frac{n*c_{s}}{2*\left(l+\Delta l+\Delta H\right)}$$

where *c_s_* is the sound velocity, *n* the mode number and *l* is the length of the cell. A correction has to be included for the open-end resonator and can be approximated as an elongation of the resonator by Δ*l* ≈ 0.6*r*, where *r* is the radius of the resonator [21]. Further, including the small hole (with diameter *d_h_*) in the resonator subtly changes the resonance frequency due to the gas flow changes and small pressure variations (Δ*H* ≈ 3*d_h_*) [22]. Given the dimensions of the cell represented in Figure 1a, the theoretical resonant frequency of the gas cell is 1.8 kHz.

As shown in Figure 1b, the resonant cell was built of aluminium in three sections (two buffers and one resonator cylinder), assembled using threads and insulated at the joints. Two fused silica high-precision windows with low-loss standard broadband antireflection coatings (Ø1/2" WG40530-C UVFS Broadband Precision Window, AR Coated: 1050–1700 nm, *t* = 3 mm, Thorlabs) were installed on both sides of the cell. The advantage of having the output window is that it allowed direct absorption detection at the same time as the acoustic detection; furthermore, it allows the implementation of a two-pass system using a mirror. The electrical microphone and diaphragm were installed in the centre of the resonator.

An electrical microphone and a thin diaphragm were used as acoustic detectors. The first was drilled through the resonator and the surface of the electrical microphone was installed flush with the resonator wall to efficiently detect the acoustic wave. The electrical microphone (FG-23329-P18, Knowles electronics) has a sensitivity of 22 mV/Pa.

The diaphragm for the optical microphone was installed in front of the electrical microphone in the external part of the resonator tube; it was fixed using a thin double-sided adhesive tape that allowed proper sealing to prevent leakage. An acoustic wave produced from a PAS generates a periodic deformation of the diaphragm, whose amplitude in the centre of the membrane (*w*_0_) can be described by Equation (2):

(2) $$w_{0}=\frac{3\;r_{m}{}^{4}\;\left(1-v_{m}{}^{2}\right)}{16\;t_{m}{}^{3}E_{m}}*Pr$$

where *r_m_* and *t_m_* are the radius and thickness of the membrane, respectively, *ν_m_* and *E_m_* are the Poisson´s ratio and Young´s modulus of the membrane material, respectively and *Pr* is the pressure generated by the acoustic wave.

A thin film (*t_m_* = 75 μm) of polytetrafluoroethylene (PTFE), also named Teflon, was used as the diaphragm material due to its physical-mechanical properties. It has a Young’s modulus of *E_m_* = 0.5 GPa and a Poisson´s ratio of 0.46 at room temperature [23]. It is considered a sensitive diaphragm for pressure transducers because of its thickness, large elastic deformation range and stiffness. Furthermore, it is extremely resistant to chemical components, external conditions such as extreme weather conditions and light and it is crucially important for its high resistance and gas permeability. The pressure sensitivity in terms of pressure-induced deflection is calculated to be 3.5 nm/Pa using Equation (2), and the data provided.

### 2.2. Interferometric Readout of the Membrane Deformation

**Figure 2:**
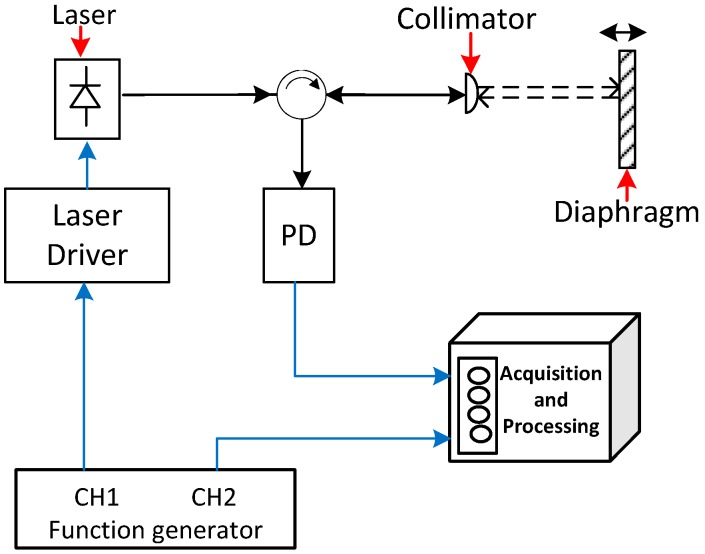
Basic scheme for the diaphragm interferometric displacement readout.

The deformation induced in the Teflon diaphragm was measured by mean of a FLDV using a CPT and phase-generated carrier demodulation [18,24]. The common-path interferometer is the optical cavity between the distal end of a normally cleaved optical fibre and the diaphragm, as represented in Figure 2. The input laser beam is transmitted to the target (Diaphragm) via a circulator and is focused onto the target using an optical collimator; the beam is diffused on the diaphragm and reflected back into the same fibre end. The second reflective layer of the interference cavity is the air/fibre interphase. The reflected light returns via the circulator to a photodiode (PD) where the interference is detected. As can be seen, the fibre acts as a patch cord to lead the light to the target and the interference signal to the detector. Therefore, disturbances on the fibre have a minimal impact on system performance based on the common path configuration. The membrane deformation can be obtained from the measured phase shift in the interferometer:

(3) $$\Delta L=\frac{\lambda }{2\pi \cdot n}\Delta \varphi$$

where *Δφ* is the optical phase shift, *n* is the refractive index of the medium in the cavity (*n_Air_* ≈ 1), *λ* is the wavelength of the single-mode laser and *ΔL* is the deflection of the membrane (i.e.,: the cavity length change due to the photo-acoustic signal).

A reflected interferometric signal is processed through a heterodyne detection [25,26] based on a phase-generated carrier (PGC) where modulation of the optical frequency of the laser forces a phase modulation in the unbalanced interferometer:

(4) $$\Delta \varphi_{c}=\frac{2\pi *n*\Delta \nu \;}{c}L$$

where *Δφ_c_* is the optical phase modulation, *Δν* is the amplitude modulation of the laser’s optical frequency, *c* is the speed of light in vacuum and *L* is the imbalance of the interferometer, that is the cavity length in this case. A practical implementation is to modulate the injection current of a diode laser at a fixed frequency (*f_c_*), which generates a carrier by phase modulation (PGC). This improves the detection accuracy of the optical phase and reduces complexity because stabilizing the interferometer is not necessary. Typically, a laser with an extremely low phase noise combined with frequency modulation capabilities is required. In the implemented PGC demodulation, the interference signal is mixed with the fundamental carrier and with the second harmonic to obtain two components in quadrature (phase shifted π/2 rad). After low-pass filtering each component, the phase signal (Δφ) can be demodulated. The signal analysis can be implemented in digital systems [27,28] to improve processing time, integration and portability.

The PGC interferometer was implemented using a 1310 nm distributed feedback (DFB) laser (BLPD-RA-70BR, Bely communication). The operating current was set to 37 mA, emitting an optical power of 10 mW, and the modulation signal to chirp the DFB laser was a 3 MHz sinusoid generated by CH1 (*f_c_*) of the signal generator (DG4162, Rigol). The laser beam was focused on the Teflon membrane (M) using adjustable aspheric collimators (F2) (focal length 4.6 mm, CFC-5X-C, Thorlabs) installed on a Ø1" Kinematic Mirror Mount 5 cm from the target. To improve the reflected signal in the target (Teflon diaphragm), the outer face of the film was sprayed with metallized micro-particles to increase the reflectivity in the same direction as the incident light beam. The interferometric signal was detected using an optical detector (PD) (PD10CFEC InGaAs Amplified Detector, BW 150 MHz, 800–1700 nm, Thorlabs) in port 3 of the circulator and a low-pass filter (LPF) (EF516, DC-4.5 MHz, Thorlabs) is installed between photodetector and acquisition system (DAQ). The reference signal was achieved using channel 2 of the function generator (Ch2) for heterodyne detection. The electric signals (Reference, reflected) were acquired and processed using a LabVIEW-controlled PXI DAQ system (NI PXIe-1071, with PXI-oscilloscope 5105, 12 bit, 60 MS/s, 8 channel), which also features synchronization capabilities, and was connected to the function generator.

### 2.3. Experimental Photoacoustic Setup

**Figure 3:**
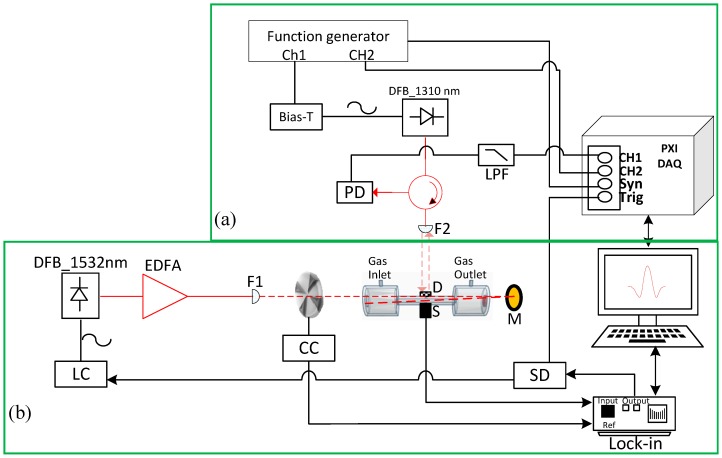
Experimental measurement setup: (**a**) PGC interferometer. DFB_1310 nm, DFB laser at 1310 nm; PD, photodetector; LPF, low pass filter; DAQ, data acquisition; and F1, F2 collimators. (**b**) Photoacoustic system. DFB_1532 nm, DFB laser at 1532 nm; LC, laser controller; EDFA, erbium-doped fibre amplifier; CC, chopper controller; D, diaphragm; S, microphone; M, mirror; SD, signal divider; Lock-in amplifier.

Photoacoustic generation is based on a classical setup, where the acoustic wave is generated by heating the molecules of the target gas, which is illuminated by a modulated pump laser tuned to the specific wavelength of absorption of the target gas. The deformation generated by the acoustic waves is detected by means of sensors installed in the gas cell, the electrical microphone and the diaphragm with interferometric displacement readout. The complete system is shown in Figure 3, where the photoacoustic set up is shown in Figure 3b and Figure 3a shows the interferometric detection described in the previous section.

A fibre coupled wavelength-stabilized laser diode with a distributed feedback laser diode (QDFBLD-1530-20, Qphotonics) emitting at 1530 nm was employed as a compact, efficient and tuneable spectroscopic source emitting with an optical power of 20 mW in the molecular fingerprint region of the NH_3_. The threshold current is approximately 10 mA. The laser has a temperature tuning coefficient of approximately 0.1 nm/ºC and current tuning coefficients of 0.011 nm/mA. The internal temperature and operating current were controlled using a compact laser diode driver (LD) with TEC and Mount for Butterfly Packages (CLD1015-Thorlabs). The laser operating point was set in the vicinity of the selected absorption line and fine tune the laser injection current allowed us to sweep the laser wavelength around a selected absorption line.

The optical signal was amplified using an erbium-doped fibre amplifier (EDFA) (CEFA-C-PB-HP, Keopsys) at a 900 mA operating current, which provides an optical power gain of approximately 6.9 dB, to obtain an optical power of 100 mW. The laser beam was focused (F1) with an adjustable aspheric collimator (CFC-8X-C, Thorlabs), The optical beam is passed through the cell two times, due to the fused silica Broadband dielectric mirror (M) (BB05-E04, Ø1/2" Broadband Dielectric Mirror, 1280–1600 nm, Thorlabs) installed at the end of the gas cell. The electrical microphone (S) and diaphragm (D) are placed in the middle of the cylinder where the maximum pressure oscillations are located. The signals from the electrical microphone were analysed by an external lock-in amplifier, then digitized and processed with the LabVIEW program.

The laser modulation to generate the acoustic signal can be done using two different methods. To calibrate the resonance frequency of the gas cell, we used amplitude modulation using an external mechanical chopper (CC in Figure 3b) (optical chopper system MC2000B with MC1F10HP chopper blade, Thorlabs) whose control signal was also the reference signal to the lock-in amplifier (SR830 lock-in amplifier, Stanford research system). For the rest of the experiments, we used wavelength modulation (WMS) of the pump laser; in this case, the lock-in amplifier generated the laser current modulation signal. By dividing the modulation signal generated by the lock-in amplifier (SD in Figure 3a), we also obtained a trigger signal that was used for sampling the PGC interferometric signal synchronously with the pump laser that excites the photo-acoustic signal.

**Figure 4:**
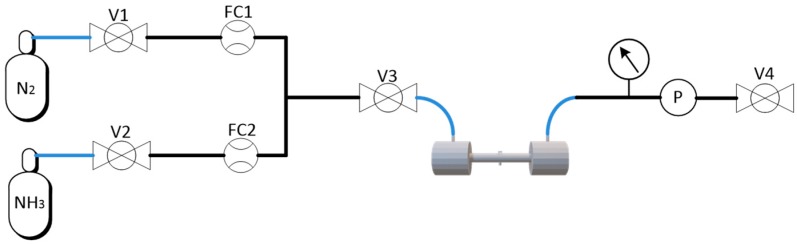
Piping, instrumentation, and safety flow diagram. V, valves; FC, Flow control; P, Pressure controller.

Different NH_3_ concentration levels within the 0–5000 ppmv range were achieved by dynamic dilution of a certified 5000 ppm NH_3_ calibration gas with ultra-high purity N_2_ using a custom-made gas mixing and conditioning system, as shown in Figure 4. It was comprised of two mass flow controllers (F-201CV, Bronkhorst) with maximums flow rates of 600 L/min (FC1) and 60 L/min (FC2). For the experiments, the pressure inside the system was kept at 1 atm, while the flow was maintained at 60 mL/min, which provided a maximum concentration uncertainty of 2.8% at 30 ppm.

A program made in LabVIEW allowed complete system control and monitoring variables such as concentration, pressure, flow, the amount of gas in the supply bottle and automatic measurements with different parameters and execution times.

## 3. Results

### 3.1. NH_3_ Wavelength Selection

**Figure 5:**
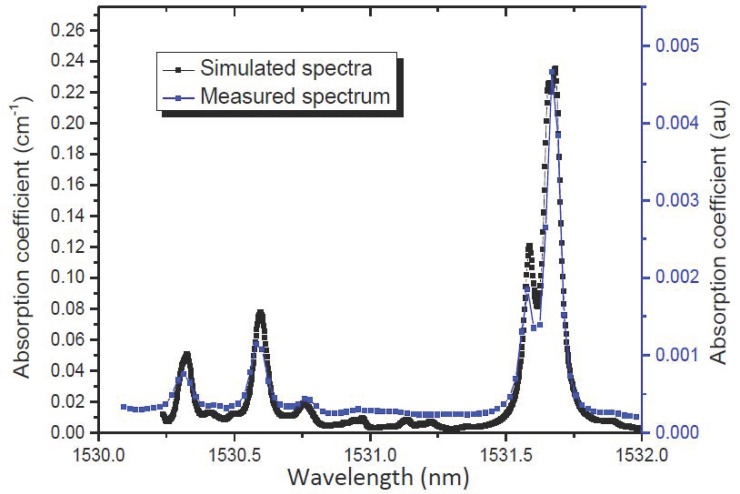
Simulation of the absorption spectra of pure NH_3_ using the HITRAN database (black line) and measured absorption spectrum (blue line). The pressure is set to 1 atm and the temperature to 296 K.

In order to identify the gas trace, a distributed feedback laser diode was employed, and the temperature and current were adjusted to obtain the maximum emitting power and specific wavelength. Coarse wavelength tuning from 1530.8 to 1532 nm at a 70 mA fixed laser current can be achieved by increasing the laser temperature from 20 to 30 °C and fine frequency tuning in the range of 0.1 nm was accomplished by changing the laser current from 90 to 100 mA. The most intense absorption band for NH_3_ in the spectral region between 1530 and 1532 nm, with the strongest line centred at 1531.68 nm. Figure 5 shows HITRAN2016 (High-resolution transmission molecular absorption database) simulated absorption coefficient for rotational–vibrational transitions in the combination band v1 + v3 and the overtone 2v3 [29] for the range chosen. The experimental identification of the absorption line was made by measuring the photoacoustic signal using the theoretical modulation frequency of 1.8 kHz and detecting the signal with the electrical microphone inside the resonant cell with 5000 ppm NH_3_. This is plotted in the same figure for current from 10 to 100 mA and at 16 to 30 °C, which is equivalent to a range of approximately 2-nm wavelength.

### 3.2. Resonant Gas Cell and Multipass Characterization

**Figure 6:**
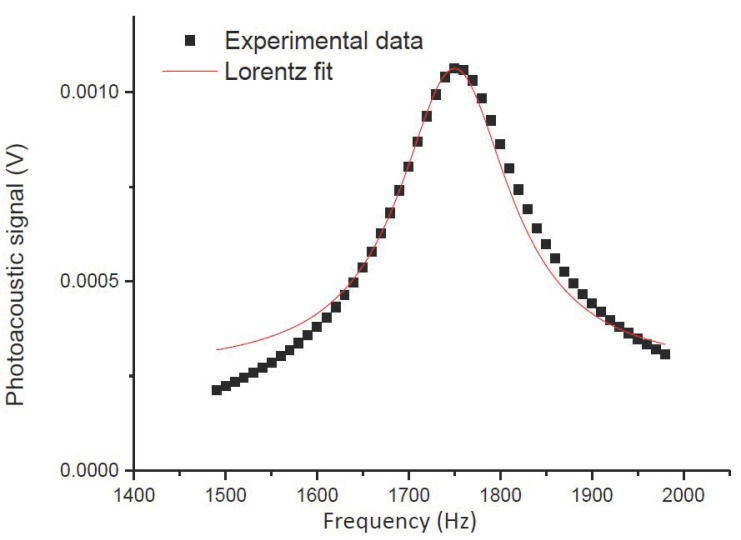
Photoacoustic signal under different modulation frequencies, from 1.5 to 2 kHz, when the laser wavelength was set at 1531.68 nm in the absorption line of NH_3_.

The resonant frequency characterization of the gas cell was accomplished using a mechanical chopper to modulate the intensity of the optical signal at different frequencies. The gas cell was filled with NH_3_ at 5000 ppm. The laser wavelength was set in the vicinity of the NH_3_ absorption line (around of 1531.7 nm), using an optical spectrum analyser to identify the wavelength and the frequency modulation was changed in the range of 1.5 to 2 kHz to examine the resonant frequency of the aluminium resonant gas cell. The frequency response is depicted in Figure 6 and the results were fitted by a Lorentzian distribution to determine the quality factor and the resonance frequency. The maximum peak for the photoacoustic signal was identified at 1755 Hz and a quality factor of 10 was obtained for the cell with the electrical microphone.

The maximum photoacoustic signal was achieved when the laser was stabilized at 26 °C and 95.4 mA, where the laser is emitting an optical power of 20.4 mW.

**Figure 7:**
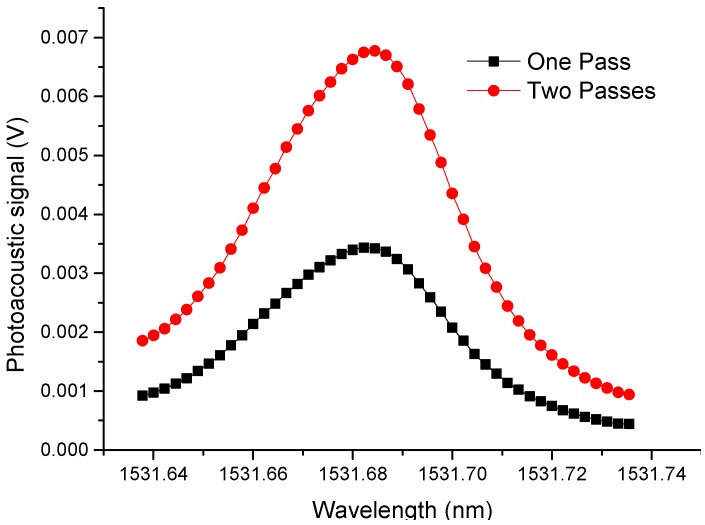
Photoacoustic signal at one optical beam pass (Black line) and two passes (red line).

To improve the detection of NH_3_, the optical beam is passed through the cell two times (filled with 3000 ppm of NH_3_) and the laser wavelength was tuned from 1531.64 nm to 1531.74 nm. The chopper frequency was set stablished at the cell resonance frequency (1.75 kHz). A mirror was set at the end of the cell to reflect the laser beam back to the cell. Laser and mirror were installed on Ø1" and Ø0.5" Kinematic Mirror Mounts, respectively, and multi-axis manual translation stages to optimize the signal with two passes. The optoacoustic signal for one and two passes are shown in Figure 7, which shows an improvement in the amplitude of the photoacoustic signal by approximately a factor of 2 [30].

### 3.3. Linearity and Sensitivity

The acoustic pressures generated inside the cell were known by means of the electrical microphone response, which was used for investigating the minimum detectable pressure. Likewise, the performance of the PAS sensor with the optical microphone was tested by intensity modulation directly and compared with electrical microphone in terms of sensitivity and linearity. Constant frequency modulation was established in the mechanical chopper at the resonant frequency of the cell (1.75 kHz) and the DFB laser emission wavelength was set to the centre of the absorption line while the concentration was changed.

**Figure 8:**
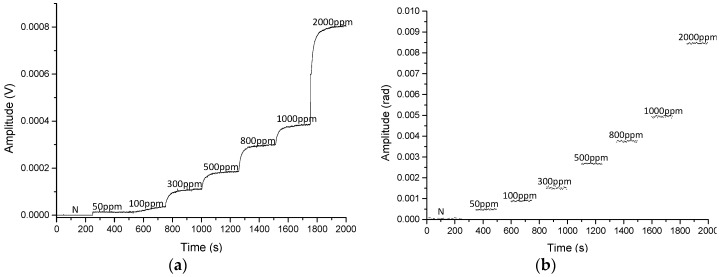
Calibration curve of 0–2000 ppm NH_3_ for electrical microphone (**a**) and optical microphone (**b**). Note that while the concentration is stabilized, the data in (b) are not represented.

The 5000 ppm calibration mixture was diluted to different concentration levels at 1 atm and a 60 mL/min constant rate flow. The PAS signal was measured by the electrical microphone and by the diaphragm interrogated with the PGC interferometer for 2000 s, as shown in Figure 8.

**Figure 9:**
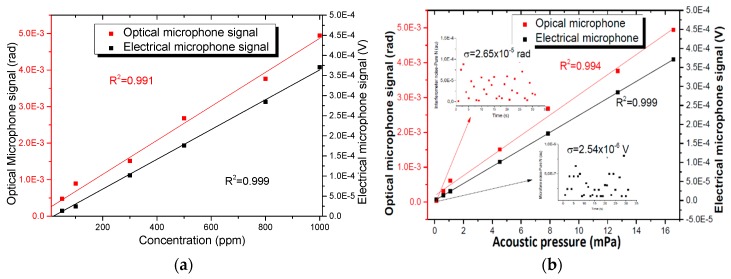
Calibration characteristics and linear fitting. (**a**) The calibration characteristics of the system ranging from 0 ppm to 2000 ppm NH_3_ diluted in N_2_. (**b**) The linearity of the two sensors versus the amplitude of the acoustic pressure wave.

The signal amplitude as a function of NH_3_ concentration is plotted in Figure 9. The calculated R^2^ value, which represents how well the regression line approximates real data points, is approximately 0.991 and 0.999 for the optical and electrical microphone, respectively. After a linear fitting procedure, excellent linearity was achieved for the two sensors. A sensitivity of 4.65 × 10^−6^ rad/ppm was obtained for the optical microphone implemented with a diaphragm and a PCG interferometer.

Different NH_3_ concentration levels (0 to 2000 ppm) generated acoustic waves with pressures from 0 to 34.6 mPa inside the gas cell, assuming the 22 mV/Pa for the electrical microphone sensitivity. Figure 9b shows the amplitude of the two sensors versus the amplitude of the acoustic pressure wave and also presents the noise measured with only N_2_. The measured output is 1.51 × 10^−3^ rad (0.2 nm) when the acoustic pressure level is 4.5 mPa (at 300 ppm) for the diaphragm, obtaining an experimental sensitivity of 40 nm/Pa. The amplitude at the same conditions for the electrical microphone was 1.01 × 10^−4^ V. The standard deviations of the noise with pure N_2_ is 2.65 × 10^−5^ rad (optical microphone) and 2.54 × 10^−6^ V (electrical microphone), which results in a minimum detectable pressure of 79.5 μPa/Hz^1/2^ [28] for a 1 Hz resolution bandwidth and of 113 μPa/ Hz^1/2^, respectively.

### 3.4. Determination of the Optimum Wavelength Modulation Depth

**Figure 10:**
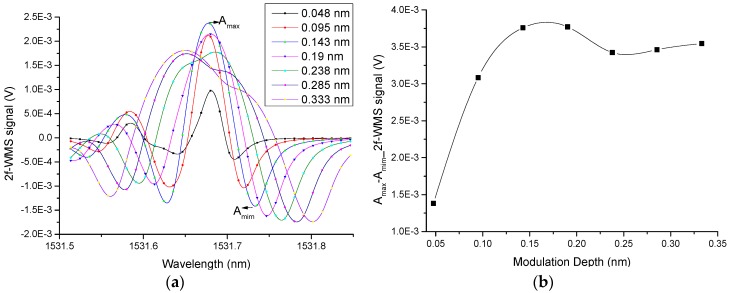
(**a**) 2f-WMS Photoacoustic signal for modulation depths from 0.048 nm to 0.333 nm of the same NH_3_ absorption lines. (**b**) Peak to peak 2f-WMS photoacoustic signal as a function of the modulation depth. All measurements were performed with a 5000 ppmv NH_3_ in N_2_ mixture, atmospheric pressure, room temperature and a 300 ms lock-in amplifier time constant.

Quantitative measurements of NH_3_ were performed using dry NH_3_ gas mixture in order to investigate the detection limit of the optical microphone. For this experiment, we generated the photoacoustic signal by wavelength modulation of the absorbing laser instead of amplitude modulation with the chopper. Wavelength modulation spectroscopy with second harmonic detection (2f-WMS) is used in this method since the generally dominating 1/f electronic noise can be drastically minimized and high detection sensitivities of NH_3_ can be achieved. A small sinusoidal signal modulates the laser wavelength directly at half the resonant frequency (fr/2 = 877 Hz). The electrical microphone sensed the photoacoustic signal and the second harmonic was detected by the lock-in amplifier. This allowed obtaining the amplitude and phase of the signal, as components X and Y. Usually, the signal is represented in component X, and the difference between the maximum (Amax) and minimum (Amin) is measured (see Amax and Amin in Figure 10a). However, the interferometric detection shows the amplitude of the signal; for this reason, these are also plotted. To establish the optimum modulation, different amplitudes of modulation (Amod) were set while the gas cell was filled with 5000 ppm NH_3_. To observe the full absorption line, the laser current was swept from 80 to 110 mA, covering a spectral bandwidth of 0.33 nm. Figure 10 shows the 2f-WMS PAS signal for modulation depths from 4.58 to 30 mA (0.048 to 0.333 nm) with steps of 4.3 mA (0.048 nm) measured with the electrical microphone (Figure 10a) and the Peak to Peak amplitude of the PAS signal (difference between maximum (Amax) and minimum (Amim)) as a function of the modulation depth (Figure 10b). This range of modulation depths corresponds to between 0.6 and 4 times the full width at half maximum (FWHM) of the absorption line (~0.082 nm). The best response was obtained with a 0.140 nm modulation depth.

### 3.5. Low Concentration Detection of NH_3_

**Figure 11:**
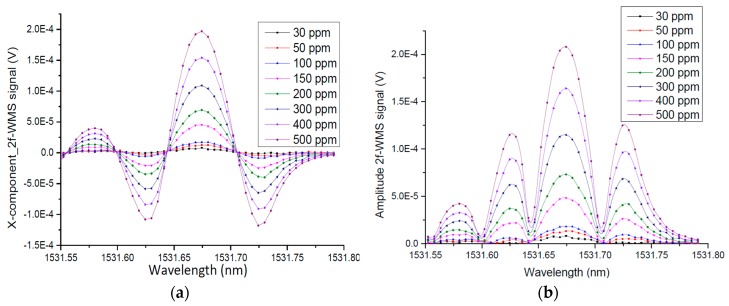

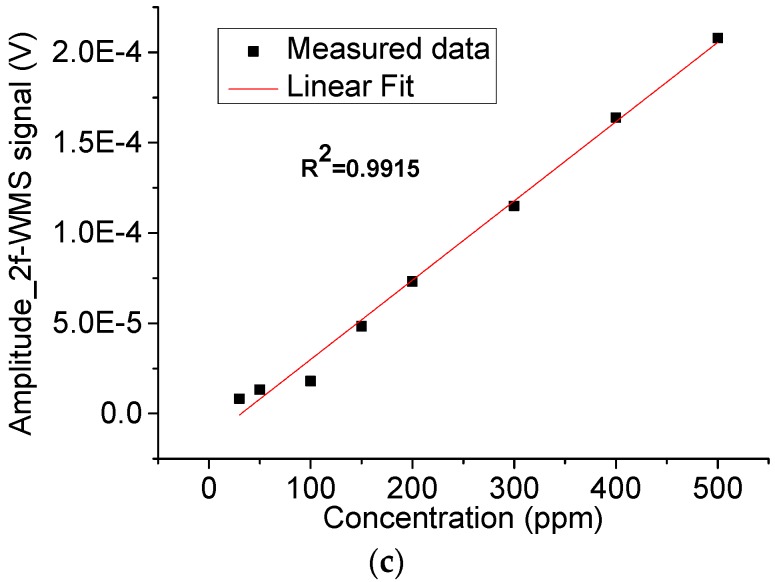
2f-WMS Photoacoustic signal measured by electrical microphone for different NH_3_ concentrations (**a**) X-component. (**b**) Amplitude and (**c**) Linear fitting. The pressure is set to 1 atm, and temperature to 296 K.

For evaluating the limit of detection (LOD) of the sensor prototype, different NH_3_ concentration levels within a range from 0 to 500 ppm were prepared by diluting a certified 5000 ppm NH_3_:N_2_ calibration mixture with 5.0 N_2_ (99.999%). Each concentration step was measured with the electrical microphone and with the optical microphone. The 2f signal was acquired and processed with a lock-in time constant of 0.3 s, a filter slope (roll-off) of 24 dB/octave, and a PXI DAQ with a 12 MHz sampling frequency, a block size of 40000 samples and averaging of 32 samples, respectively. The measured 2f-WMS signal (Amplitude and x-component) corresponding to different NH_3_ concentration levels and the linearity plot of the microphone sensors are demonstrated in Figure 11. The R^2^ value to experimental data is 0.992.

**Figure 12:**
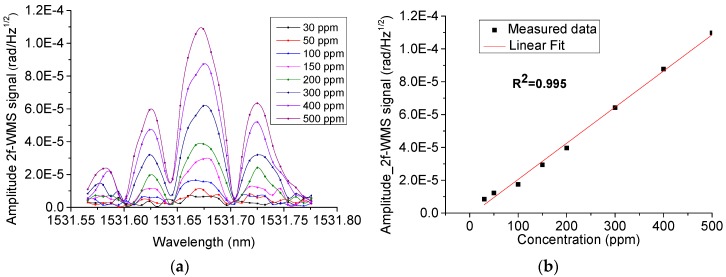
Amplitude (**a**) and linear fitting (**b**) of 2f-WMS Photoacoustic signal measured by optical microphone for different NH_3_ concentrations. The pressure is set to 1 atm, and the temperature to 296 K.

The resulting 2f-WMS amplitude curve normalized to the bandwidth detected by the optical microphone is plotted in Figure 12. Good linearity is observed between signal and NH_3_ concentrations for the optical microphone (R^2^ = 0.995).

**Figure 13:**
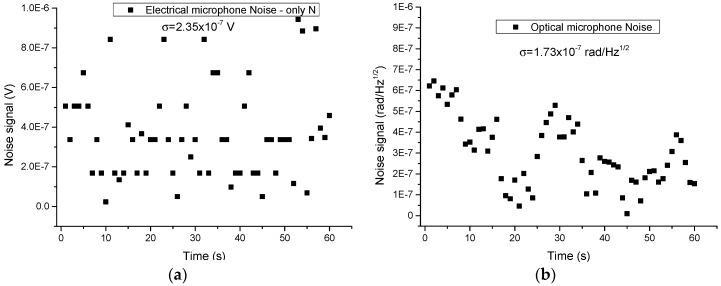
Noise signal for the acquired 2f-WMS photoacoustic signal for pure N_2_. (**a**) Electrical microphone and (**b**) Optical microphone.

To estimate the noise, the excitation laser central wavelength was tuned to 1531.7 nm and the noise level was determined from the baseline recorded with the gas cell filled with N_2_. The lock-in and the interferometer outputs were then sampled at a rate of 1 sample per second for 60 s, as shown in the inset of Figure 13. From this data set, we can conclude that the standard deviation of the noise sample (1σ) is 2.35 × 10^−7^ V and 1.73 × 10^−7^ rad/Hz^−1/2^, which combined with the peak-to-peak signals data obtained at 30 ppm of NH_3_ concentration, leads to a signal-to-noise ratio (SNR) of 35 and 49 for the electrical and optical microphone, respectively.

The minimum detectable NH_3_ concentration level (SNR = 1) by electrical microphone sensor was estimated to be 535 ppb for a time constant of 0.3 s. Hence, the corresponding normalized noise equivalent absorption (NNEA) coefficient for NH_3_ was 2.48 × 10^−8^ W cm^−1^ Hz^−1/2^ obtained based on the corresponding detector bandwidth of 0.26 Hz. A minimum detection limit (SNR = 1) of 785 ppb and an NNEA = 1.85 × 10^−8^ W cm^−1^ Hz^−1/2^ using Hanning window functions on fast Fourier transformation (FFT) [31] were obtained for the optical microphone.

## 4. Conclusions

A high-sensitivity optical microphone based on a diaphragm and phase interferometer was demonstrated for NH_3_ detection. A thin membrane with low Young’s modulus results in an excellent acoustic wave transducer. A pigtailed, near infrared, continuous wave DFB diode laser emitting at 1.53 μm was employed as the laser excitation source. Furthermore, we designed a resonant gas cell to improve the acoustic signal and the behaviour of the system.

The detection based on a fibre laser Doppler vibrometer using a common path topology with PGC demodulation proved to be an excellent configuration, easy to implement and versatile, which allows obtaining good linearity and sensitivity in combination with a polymer membrane. This configuration also reduces complexity because the interferometer laser does not need to be stabilized. We obtained the minimum detectable pressure of 79.5 μPa/Hz^1/2^, which is similar to other systems in the literature, and this can be improved using smaller Young´s modulus polymers and changing the physical dimensions. In addition, the common-path non-contact interferometric read-out is easily reconfigurable to different sizes of the gas cell and of the membrane.

Parts per billion (785 ppb) were measured by this configuration when the modulation depth was adjusted to the optimum value of 0.14 cm^−1^. The normalized noise equivalent absorption coefficient (1.85 × 10^−8^ W cm^−1^ Hz^−1/2^) measured using an optical microphone with interferometric detection has the same order to the conventional PA results, such as the systems that used commercially available cantilever-enhanced photoacoustic detection [13] and QEPAS-based NH_3_ sensor in the near infrared [32]. The sensitivity performance of this photoacoustic sensor based on the PGC interferometer can be further increased by optimizing the resonant cell (Q-factor and multipass). Further studies are focused in the miniaturization of the cell and the mechanical transducer, as the PGC interferometer is readily designed to work well above acoustic frequencies. The photoacoustic sensor with optical microphone can be used for gas detection in hazardous environments or where humidity, high temperature or strong electromagnetic interferences are presented.

## References

[1] Harren F.J.M., Mandon J., Cristescu S.M. (2012). Photoacoustic Spectroscopy in Trace Gas Monitoring. Encycl. Anal. Chem..

[2] Khush R.S., Arnold B.F., Srikanth P., Sudharsanam S., Ramaswamy P., Durairaj N., London A.G., Ramaprabha P., Rajkumar P., Balakrishnan K. (2013). H_2_S as an Indicator of Water Supply Vulnerability and Health Risk in Low-Resource Settings: A Prospective Cohort Study. Am. J. Trop. Med. Hyg..

[3] Bozóki Z., Pogány A., Szabó G. (2011). Photoacoustic Instruments for Practical Applications: Present, Potentials, and Future Challenges. Appl. Spectrosc. Rev..

[4] Ma Y. (2018). Review of Recent Advances in QEPAS-Based Trace Gas Sensing. Appl. Sci..

[5] Xu F., Ren D., Shi X., Li C., Lu W., Lu L., Lu L., Yu B. (2012). High-Sensitivity Fabry – Perot Interferometric Pressure Sensor Based on a Nanothick Silver Diaphragm. Opt. Lett..

[6] Akkaya O.C., Kilic O., Digonnet M.J.F., Kino G.S., Solgaard O., Engineering E. High-Sensitivity Thermally Stable Acoustic Fiber Sensor. Proceedings of the IEEE 2010 Sensors.

[7] Ma J., Jin W., Ho H.L., Dai J.Y. (2012). High-Sensitivity Fiber-Tip Pressure Sensor with Graphene Diaphragm. Opt. Lett..

[8] Wang Q., Wang J., Li L., Yu Q. (2011). An All-Optical Photoacoustic Spectrometer for Trace Gas Detection. Sens. Actuators B Chem..

[9] Tan Y., Zhang C., Jin W., Yang F., Ho H.L., Ma J. (2017). Optical Fiber Photoacoustic Gas Sensor with Graphene Nano-Mechanical Resonator as the Acoustic Detector. IEEE J. Sel. Top. Quantum Electron..

[10] Cao Y., Jin W., Ho H.L., Ma J. (2013). Miniature Fiber-Tip Photoacoustic Spectrometer for Trace Gas Detection. Opt. Lett..

[11] Lindley R.E., Parkes A.M., Keen K.A., Mcnaghten E.D., Orr-ewing A.J. (2007). A Sensitivity Comparison of Three Photoacoustic Cells Containing a Single Microphone, a Differential Dual Microphone or a cantilever pressure sensor. Appl. Phys. B.

[12] Kauppinen J., Wilcken K., Kauppinen I., Koskinen V. (2004). High Sensitivity in Gas Analysis with Photoacoustic Detection. Microchem. J..

[13] Moser H., Lendl B. (2016). Cantilever-Enhanced Photoacoustic Detection of Hydrogen Sulfide (H_2_S) Using NIR Telecom Laser Sources near 1.6 µm. Appl. Phys. B Lasers Opt..

[14] Hirschmann C.B., Lehtinen J., Uotila J., Ojala S., Keiski R.L. (2013). Sub-Ppb Detection of Formaldehyde with Cantilever Enhanced Photoacoustic Spectroscopy Using Quantum Cascade Laser Source. Appl. Phys. B Lasers Opt..

[15] Gruca G., Heeck K., Rector J., Iannuzzi D. (2013). Demonstration of a Miniature All-Optical Photoacoustic Spectrometer Based on Ferrule-Top Technology. Opt. Lett..

[16] Gorelik A.V., Starovoitov V.S. (2017). Demonstration of a Highly Sensitive Photoacoustic Spectrometer Based on a Miniaturized All-Optical Detecting Sensor. Opt. Express.

[17] Lee B.H., Kim Y.H., Park K.S., Eom J.B., Kim M.J., Rho B.S., Choi H.Y. (2012). Interferometric Fiber Optic Sensors. Sensors.

[18] Posada-Roman J.E., Jackson D.A., Garcia-Souto J.A. (2016). Variable Configuration Fiber Optic Laser Doppler Vibrometer System. Photonic Sens..

[19] Bonilla-Manrique O.E., Moser H., Martín-Mateos P., Lendl B., Ruiz-Llata M. (2019). Hydrogen Sulfide Detection in the Midinfrared Using a 3D-Printed Resonant Gas Cell. J. Sensors.

[20] Bauer R., Stewart G., Johnstone W., Boyd E., Lengden M. (2014). 3D-Printed Miniature Gas Cell for Photoacoustic Spectroscopy of Trace Gases. Opt. Lett..

[21] Miklós A., Hess P., Bozóki Z. (2001). Application of Acoustic Resonators in Photoacoustic Trace Gas Analysis and Metrology. Rev. Sci. Instrum..

[22] Koskinen V., Fonsen J., Roth K., Kauppinen J. (2008). Progress in Cantilever Enhanced Photoacoustic Spectroscopy. Vib. Spectrosc..

[23] Rae P.J., Dattelbaum D.M. (2004). The Properties of Poly(Tetrafluoroethylene) (PTFE) in Compression. Polymer (Guildf)..

[24] Tayag T.J., Watson R.C. (2012). Digital Demodulation of Interferometric Signals. Modern Metrology Concerns, Cocco, L., Ed..

[25] Zhang W., Gao W., Huang L., Mao D., Jiang B., Gao F., Yang D., Zhang G., Xu J., Zhao J. (2015). Optical Heterodyne Micro-Vibration Measurement Based on All-Fiber Acousto-Optic Frequency Shifter. Opt. Express.

[26] Krzempek K., Dudzik G., Abramski K., Wysocki G., Jaworski P., Nikodem M. (2018). Heterodyne Interferometric Signal Retrieval in Photoacoustic Spectroscopy. Opt. Express.

[27] Feng L., He J., Duan J.Y., Li F., Liu Y.L. Implementation of Phase Generated Carrier Technique for FBG Laser Sensor Multiplexed System Based on Compact RIO. Proceedings of the 2008 1st Asia-Pacific Optical Fiber Sensors Conference.

[28] Connelly M.J. (2002). Digital Synthetic-Heterodyne Interferometric Demodulation. J. Opt. A Pure Appl. Opt..

[29] Claps R., Englich F.V., Leleux D.P., Richter D., Tittel F.K., Curl R.F. (2001). Ammonia Detection by Use of Near-Infrared Diode-Laser-Based Overtone Spectroscopy. Appl. Opt..

[30] Ma Y., Qiao S., He Y., Li Y., Zhang Z., Yu X., Tittel F.K. (2019). Highly Sensitive Acetylene Detection Based on Multi-Pass Retro-Reflection-Cavity-Enhanced Photoacoustic Spectroscopy and a Fiber Amplified Diode Laser. Opt. Express.

[31] Heinzel G., Rudiger A., Schilling R. Spectrum and Spectral Density Estimation by the Discrete Fourier Transform (DFT), Including a Comprehensive List of Window Functions and Some New at-Top Windows. https://pure.mpg.de/pubman/faces/ViewItemOverviewPage.jsp?itemId=item_152164.

[32] Ma Y., He Y., Tong Y., Yu X., Tittel F.K. (2017). Ppb-Level Detection of Ammonia Based on QEPAS Using a Power Amplified Laser and a Low Resonance Frequency Quartz Tuning Fork. Opt. Express.

